# FGF19 increases mitochondrial biogenesis and fusion in chondrocytes via the AMPKα-p38/MAPK pathway

**DOI:** 10.1186/s12964-023-01069-5

**Published:** 2023-03-13

**Authors:** Shiyi Kan, Caixia Pi, Li Zhang, Daimo Guo, Zhixing Niu, Yang Liu, Mengmeng Duan, Xiahua Pu, Mingru Bai, Chenchen Zhou, Demao Zhang, Jing Xie

**Affiliations:** 1 Lab of Bone and Joint Disease, State Key Laboratory of Oral Diseases, West China Hospital of Stomatology, Sichuan University, Chengdu, 610064 Sichuan China; 2 National Clinical Research Center for Oral Diseases, West China Hospital of Stomatology, Sichuan University, Chengdu, 610064 China

**Keywords:** FGF19, Chondrocyte, Mitochondrial biogenesis, Mitochondrial fission–fusion, p38/MAPK signaling

## Abstract

Fibroblast growth factor 19 (FGF19) is recognized to play an essential role in cartilage development and physiology, and has emerged as a potential therapeutic target for skeletal metabolic diseases. However, FGF19-mediated cellular behavior in chondrocytes remains a big challenge. In the current study, we aimed to investigate the role of FGF19 on chondrocytes by characterizing mitochondrial biogenesis and fission–fusion dynamic equilibrium and exploring the underlying mechanism. We first found that FGF19 enhanced mitochondrial biogenesis in chondrocytes with the help of β Klotho (KLB), a vital accessory protein for assisting the binding of FGF19 to its receptor, and the enhanced biogenesis accompanied with a fusion of mitochondria, reflecting in the elongation of individual mitochondria and the up-regulation of mitochondrial fusion proteins. We then revealed that FGF19-mediated mitochondrial biogenesis and fusion required the binding of FGF19 to the membrane receptor, FGFR4, and the activation of AMP-activated protein kinase alpha (AMPKα)/peroxisome proliferator-activated receptor-gamma coactivator 1 alpha (PGC-1α)/sirtuin 1 (SIRT1) axis. Finally, we demonstrated that FGF19-mediated mitochondrial biogenesis and fusion was mainly dependent on the activation of p-p38 signaling. Inhibition of p38 signaling largely reduced the high expression of AMPKα/PGC-1α/SIRT1 axis, decreased the up-regulation of mitochondrial fusion proteins and impaired the enhancement of mitochondrial network morphology in chondrocytes induced by FGF19. Taking together, our results indicate that FGF19 could increase mitochondrial biogenesis and fusion via AMPKα-p38/MAPK signaling, which enlarge the understanding of FGF19 on chondrocyte metabolism.

**Video Abstract**

**Supplementary Information:**

The online version contains supplementary material available at 10.1186/s12964-023-01069-5.

## Introduction

Mitochondria play a vital role in chondrocyte metabolism because they not only provide the indispensable adenosine triphosphate (ATP) for chondrocytes [1] but also directly participate in many cellular physiological activities by changing their biogenesis [2]. The homeostasis of mitochondrial biogenesis is maintained by a mitochondrial quality control (MQC) system [3]. MQC mainly preserves functional mitochondria by controlling the homeostasis of the fission–fusion process, and even removes redundant non-functional mitochondria [4]. Mitochondrial fission is mainly driven by dynamin-related protein 1 (Drp1), a cytoplasmic dynamin guanosine triphosphatase (GTPase) [5], and mitochondrial fission protein 1 (Fis1) [6]. Drp1 is dynamically recruited to the outer mitochondrial membrane (OMM) and then oligomerized in a ring-like structure and drives membrane constriction in a GTP-dependent manner. Fis1 serves as a membrane-anchor that could also regulate mitochondrial fission through interaction with Drp1 and other fission components in mitochondria [7]. For mitochondrial fusion, it is controlled by 2 mitofusins (Mfn1 and Mfn2) [8] and dominant optic atrophy 1 (Opa1) [9]. Mfn1 and Mfn2 mediate the fusion of OMMs and then Opa1 mediates the fusion of the inner mitochondrial membrane (IMM). The outer membranes of two mitochondria are tethered by Mfns. GTP binding and/or hydrolysis induces a conformational change of Mfns, resulting in increased mitochondrial docking and membrane contact sites. Following OMM fusion, the interaction between OPA1 and cardiolipin (CL) on either side of the membrane tethers the IMMs, which drives IMM fusion by OPA1-dependent GTP hydrolysis. Mitochondrial fusion promotes the exchange of important components among mitochondria, especially mitochondrial deoxyribonucleic acid (mtDNA), and ensures the continuity of mitochondrial function [10]. Mitochondria adjust their number and mitochondrial network morphology in cells by coordinating the cycle of mitochondrial fission and fusion. These dynamic changes further regulate mitochondrial functions and determine cell metabolism [11].

Fibroblast growth factors (FGFs) are a type of cytokine that plays an important role in regulating organic growth, development, maturation and disease [12]. They include a total of 22 members of 7 subfamilies. FGF19, belonging to the FGF19 subfamily including FGF19, FGF21 and FGF23, was first found to be expressed in the human cartilage in 1999 [13] and then it is recognized to be one of the predominant FGF ligands present in developing human cartilage [14]. These reports indicate its potential role in chondrocyte development and homeostasis. Previous evidence has confirmed that FGF19 signalling is crucial to glucose metabolism [15]. It could increase energy consumption and glucose utilization by increasing the cyclic-AMP response binding protein (CREB)-peroxisome proliferator-activated receptor-gamma coactivator 1 alpha (PGC-1α)-signalling cascade. Previous studies have shown that mitochondria are the potential targets of FGF19. FGF19 has been shown to increase energy homeostasis by increasing fatty acid delivery to mitochondria in the liver [16]. In white adipose tissue, FGF19 levels are correlated with the mitochondrial number [17]. FGF19 can prevent excessive palmitate-induced dysfunction of differentiated mouse myoblast cells by protecting mitochondrial function [18]. These results suggest that FGF19 may work as a potential mediator of mitochondrial metabolism. Besides, the receptors of FGF19 include fibroblast growth factors receptor 1c, 2c, 3c and 4 (FGFR1c, 2c, 3c & 4), but FGFR4 is considered to be the primary receptor due to its high affinity for FGF19 [19]. Report also indicates the binding of FGF19 to its receptor FGFR4 requires the participation of β-Klotho (KLB), a co-receptor to achieve high affinity [20]. FGF19 has been reported to form a dimer with the β-Klotho monomer via its C-terminal tail at 1:1 ratio [21]. Till now, although we realize the importance of FGF19 in the development and maturation of cartilage, there is a lack of evidence that FGF19 regulates cartilage behavior, especially mitochondrial changes.

Cartilage is a special structure composed of dense extracellular matrix (ECM), mainly including type II collagen and proteoglycan, and highly differentiated cells called chondrocytes [22]. In general, chondrocytes are localized in a relatively low-oxygen environment that energy producing is vital for them. Mitochondrial dysfunction could break the balance between glycolysis and oxidative phosphorylation (OXPHOS) in chondrocytes, reducing ATP production substantially [23]. Thus, in the current study, we aim to explore the effect of FGF19 on the mitochondrial fission–fusion process in chondrocytes by characterizing the morphology of the mitochondria network and its fission–fusion mediator proteins, and its underlying bio-mechanism.

## Methods and materials

### Chondrocyte isolation

The tissue materials used in the current study were obtained according to ethical principles and the protocol was firstly approved before the experiments began by our Institutional Review Board (No. WCHSIRB-OT-2020-048). Chondrocytes were isolated from 0 to 3 days’ newborn C57 mice as previously described [24]. In brief, the chondrocytes from cartilage of the knee joints were collected by 0.25% trypsin digestion for 30 min at 37 °C and 0.2% type II collagenase (No. C6885, Sigma, MO, USA) digestion for about 16–18 h at 37 °C till the cartilage tissue mass was completely digested. The isolated chondrocytes were filtered and cultured in 10% FBS DMEM (No. D6429, HyClone, Logan, UT, USA). We used the chondrocytes at passage 1–2.

### ATP assay

ATP concentrations were tested with enhanced ATP assay kit (No. S0027, Beyotime, Shanghai, China) according to the manufacturer’s protocol as previously described [25]. Cells were lysed with ATP lysis buffer (200 μl of lysate per well in 6-well plates) and centrifuged at 15, 000 g for 5–10 min at 4 °C. The lysates were collected and stored at -20℃. Before the ATP test, 100 μl ATP working solution (ATP test solution: ATP test dilution = 1: 5) was added to 1.5 ml EP tubes and incubated for 3–5 min at room temperature (RT). Next, the lysates were transferred to 100 μl of ATP working solution and mixed quickly. The amount of luminescence emitted was measured with a luminometer (Synergy HTX Multi-Mode Microplate reader, BioTek Instrument, WI, USA) immediately. The luminescence data were normalized to the control sample protein amounts. The statistical program GraphPad Prism 8 was used to process the data and images.

### Mitochondrial staining of living cells

Cell Navigator™ Mitochondrion Staining Kit (No.22667, AAT Bioquest, CA, USA) was used to stain mitochondria in living chondrocytes. Briefly, cells were cultured in petri dishes specified for confocal laser microscopy (1,000 cells per dish, Glass Bottom Cell Culture Dish, Φ15mm, No. 801002, NEST, Jiangsu, China). FGF19 (200 ng/ml, No.100-32, PEPRO TECH, USA) and/or KLB (200 ng/ml, 2619-KB-050, R&D Systems, USA) at a 1:1 ratio were added into the culture media as the experimental group and continued to incubate for 72 h. Thaw all the components of Cell Navigator™ Mitochondrion Staining Kit at RT before starting the experiment. 2 µl of 500X Mitolite™ Orange (Component A) was added into 1 ml of Live Cell Staining Buffer (Component B) to make a working solution. 200 µl working solution was added to the petri dishes and incubate at 37 °C for 30–120 min. Fluorescence was detected at Ex/Em = 540/590 nm (TRITC filter set). Replace the dye loading solution with phosphate-buffered saline (PBS, 1 ×). Then the cells were fixed in 4% paraformaldehyde for 20 min and rinsed with PBS for three times. After being penetrated by 0.5% Triton X-100 (Beyotime, Shanghai, China) for 15 min. Nuclei were counterstained with 4′,6-diamidino-2-phenylindole (DAPI; D9542, Sigma, USA) and the cytoskeleton was stained with phalloidin (FITC, A12379, Thermo, MA, USA). The immunofluorescence images were observed through a confocal laser scanning microscope (FV3000, Olympus, Tokyo, Japan).

### RNA sequencing and bioinformatics analysis

Chondrocytes (at 1 × 10^6^ cells per well) were treated by FGF19 at 200 ng/ml in the presence of KLB (200 ng/ml) for 72 h and harvested by trypsin digestion. Then the cells were sent for RNA sequencing at Shanghai Lifegenes Biotechnology CO., Ltd (Shanghai, China) as previously described [26]. Total RNA was extracted from chondrocytes using Trizol reagent (Catalog#15596026, Thermo Fisher Scientific, Waltham, MA), and the quantification was performed with an RNA Nano 6000 assay kit (Bioanalyzer 2100 System, Agilent Technologies, CA). Illumina NeoPrep system was applied to purify and fragment the mRNAs, synthesize cDNAs, and amplify the targets. Sequencing was accomplished with the Illumina NovaSeq 6000 platform, and the raw data were obtained by matching reference genome using HISAT2 v2.1.0. The data were reported in Fragments Per Kilobase of exon model per Million mapped fragments (FPKM). Pheatmap was generated by online R package.

### Transmission electron microscopy (TEM)

The cell pellets in agarose piece were treated with 1% OsO4 solution for 1 h at 4 °C, helping to provide an enhanced contrast to TEM images. Samples were further processed for dehydration, infiltration and embedding into LX-112 resin with serial changes into following solutions: 25% ethanol at RT for 15 min, 50% ethanol at RT for 15 min, 75% ethanol at RT for 15 min, 95% ethanol at RT for 15 min, 100% ethanol at RT for 15 min, twice; Ethanol: LX-112 (3:1) at RT for 30 min, Ethanol: LX-112 (1:1) at RT for 30 min, Ethanol: LX-112 (1:3) at RT for 30 min; pure LX-112 at RT for 60 min, twice. Finally, the samples were transferred in pyramid tip mold (Ted Pella; 10585) and polymerized at 60 °C for 72 h. Semi-thin sections (1 μm) were cut using an ultra-microtome (Leica EM UC7) after attaching the pyramid on mounting cylinders (Ted Pella; 10580) and stained with toluidine blue to identify the position of cells. Ultra-thin sections (70–100 nm) were cut and collected on 200 mesh grids. The grids were stained with 1% uranyl acetate, at RT for 10 min, followed by Reynolds lead citrate, at RT for 5 min. Sections were examined with a JEM-1400FLASH electron microscope (JEM-1400FLASH, JEOL, Tokyo, Japan), at 80 kV, using the AMT-600 image capture engine software. Images were transferred to photoshop software for final processing.

### Western blotting

The specific procedure followed our published paper [27]. Briefly, cells at 5 × 10^5^ per well (six-well plate) were lysed as one amount of a sample. Equal amounts of protein extracts were separated on 10% SDS-polyacrylamide gel electrophoresis, and then transferred into a PVDF membrane (IPVH00010, Millipore, Massachusetts, USA) at 200 mA for 2 h. PVDF membranes were blotted with 5% skim milk for 1 h and then the blots were probed overnight with primary antibodies overnight at 4 °C (mouse anti-β-actin, 1:1,000, sc-47778, Santa Cruz Biotechnology, Santa Cruz, USA; rabbit anti- citrate synthase (CS), 1:1000, No.383932, ZEN BIO, Chengdu, China; rabbit anti-AMPKα-1, 1:1,000, No.380431, ZEN BIO, Chengdu, China; rabbit anti-phospho-AMPK alpha 1 (Ser496), 1:1,000, No.R26252, ZEN BIO, Chengdu, China; rabbit anti-PGC-1α:1,000, No.381615, ZEN BIO, Chengdu, China; rabbit anti-SIRT1, 1:1,000, No.R25721, ZEN BIO, Chengdu, China; rabbit anti-Mfn1, 1:1,000, No.509880, ZEN BIO, Chengdu, China; rabbit anti-Mfn2, 1:1000, No.340604, ZEN BIO, Chengdu, China; rabbit anti-Opa1, 1:1000, No.382025, ZEN BIO, Chengdu, China; rabbit anti-ERK1/2, 1:1,000, ab17942, Abcam, Cambridge, UK; rabbit anti-p-ERK1/2, 1:1,000, No.4370, Cell Signaling Technology, Boston, USA; rabbit anti-p38/MAPK, 1:1,000, No.340697, ZEN BIO, Chengdu, China; rabbit anti-pp38 (Thr180/Tyr182), 1:1,000, No.9211, Cell Signaling Technology, Boston, USA; rabbit anti-JNK, 1:1,000, No.R22866, ZEN BIO, Chengdu, China; rabbit anti-phospho-JNK (Thr183/Tyr185), 1:1,000, No.340810, ZEN BIO, Chengdu, China). Membranes were washed with TBST, and the homologous secondary antibody (anti-mouse, m-IgGКBP-HRP, 1:5,000, sc-516102; anti-rabbit, IgG-HRP, 1:5,000, sc-2357, Santa Cruz Biotechnology, Santa Cruz, USA) was incubated with the membrane for 2 h. The Immobilon ® Western (P90719, Millipore, Massachusetts, USA) kit was used to visualize immune complexes, and the protein expression levels were analysed with Image J software (NIH). β-actin was used as an internal control.

### RNA extraction and quantitative real-time polymerase chain reaction (qPCR)

Total RNA was extracted from the chondrocytes using RNeasy Plus mini kit (No. 73404, Qiagen, Shanghai, China) and then reverse-transcribed to complementary DNA (cDNA) using a first-strand cDNA synthesis kit (K1621, Thermo, MA, USA) according to manufacturer’s instruction. The SYBR Premix Ex Taq II PCR Kit (RR820A, TAKARA, Dalian, China) was used to perform qPCR on an ABI 7300 instrument (Applied Biosystems, Shanghai, China). Primer sequences were designed with basic local alignment search tool (BLAST), and the sequences were as follows: glyceraldehyde-3-phosphate dehydrogenase (GAPDH), 5′-AGGTTGTCTCCTGCGACTTCA-3′ (forward) and 5′-CCAGGAAATGAGCTTGACAAA-3′ (reverse); FGFR4, 5′-AAGGTGGTCAGTGGGAAGTCTG-3′ (forward) and 5′-CAGAGGCCTCAAGGGACAAAG-3′ (reverse). Each gene sample was repeated based on three copies. The cycle threshold (CT) values were normalized to GADPH and calculated using the 2^−∆∆Ct^ method.

### Immunofluorescence and confocal laser scanning microscopy (CLSM)

Immunofluorescence staining was performed as previously described [28]. Briefly, Cells were cultured in petri dishes specified for confocal laser microscopy for 12 h. Then FGF19 (200 ng/ml) and/or KLB (200 ng/ml) were added to the culture media as the experimental group and continued to incubate for 24 h. Then the cells were fixed in 4% paraformaldehyde for 20 min and rinsed with PBS for three times. After being penetrated by 0.5% Triton X-100 for 15 min, the samples were blocked with 1% bovine serum albumin (BSA) for 1 h. Cells were then incubated with the antibodies (rabbit anti-CS, 1:1000; rabbit anti-phospho-AMPK alpha 1 (Ser496), 1:1,000; rabbit anti-PGC-1α, 1:1,000; rabbit anti-Mfn2, 1:1,000; rabbit anti-Opa1, 1:1,000; rabbit anti-pp38 (Thr180/Tyr182), 1:1,000) overnight at 4 °C. The secondary antibody was Alexa Fluor® 647 (10 μg/ml, Alexa Fluor ®647, Life Technology, Grand Island, NY, USA). Nuclei were counterstained with DAPI and the cytoskeleton was stained with FITC. Two different antibodies, conjugated with two different fluorochromes (i.e., Alexa Fluor® 488 antibody: green fluorescence; Alexa Fluor® 647 antibody: red fluorescence) were used in double-fluorescence labeling. The immunofluorescence images were observed through a confocal laser scanning microscope FV3000.

### Inhibitor treatments

P38 pathway inhibitor SB203580 (No. A8254, APExBIO Technology, TX, USA) was prepared in dimethyl sulfoxide (DMSO) (No.196055, MP Biomedicals, OH, USA) as stock solutions and the treatment procedure was followed our previous report [29]. Chondrocytes were pre-incubated by SB203580 (10 µM) for 2 h prior to the addition of FGF19. DMSO was added to the cell culture medium as a control.

### Statistical analysis

All protein bands and immunofluorescence images were quantified using optical density (OD) value and fluorescent intensity by ImageJ software (ImageJ2, NIH, Bethesda, MD, USA). Data were presented as the mean ± SD of at least three independent experiments (n ≥ 3) and plotted with Graph Pad Prism. The significant difference analyses were all based on Student T-test. In each analysis, the critical significance level was set to be *p* = 0.05.

## Results

### FGF19 increases the mitochondrial biogenesis

**Fig. 1:**
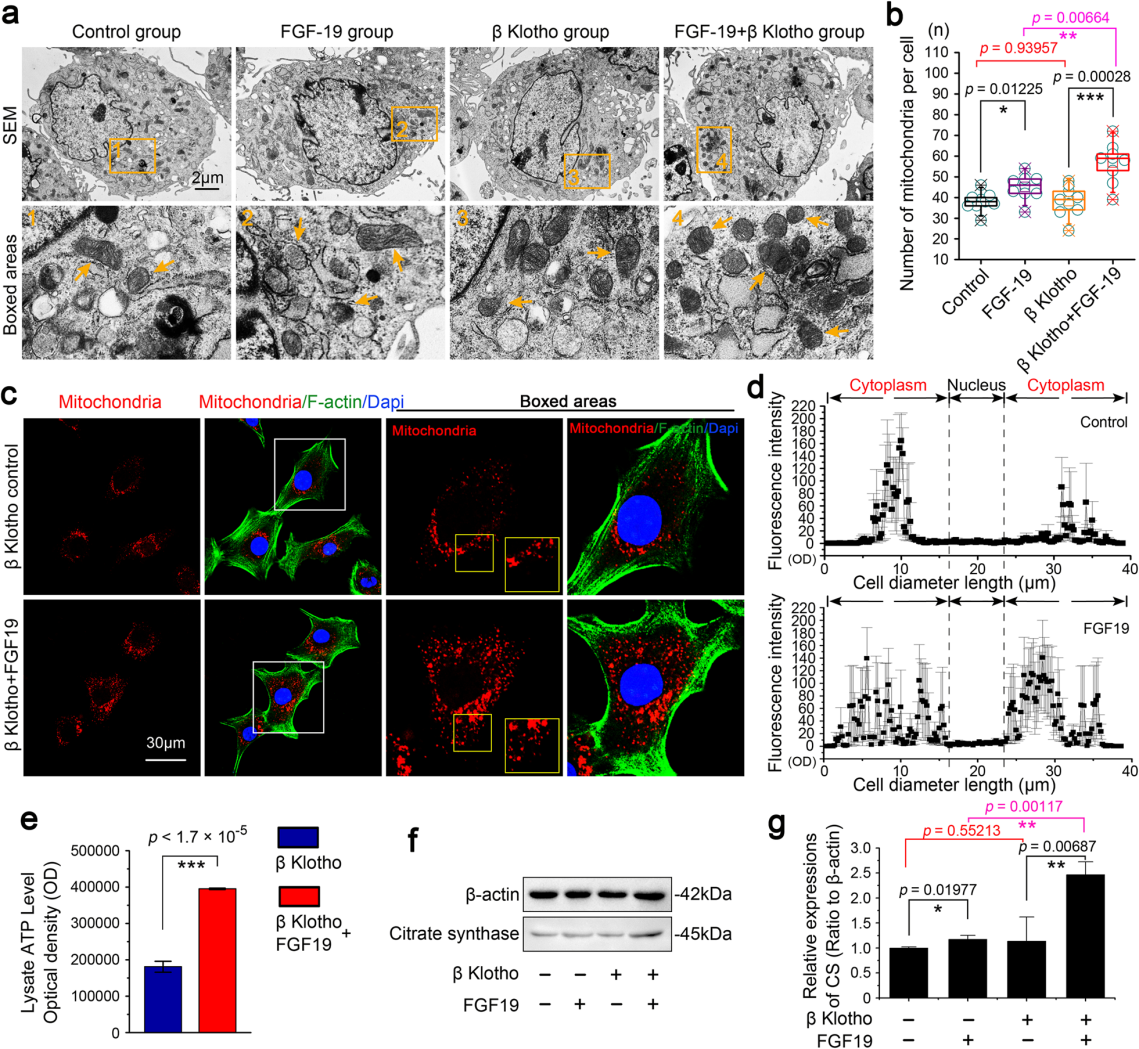
FGF19 induces a transient increase in mitochondrial number and an enhanced generation of ATP products. **a** Representative TEM images showing the changes of mitochondrial number in chondrocytes induced by FGF19 at 200 ng/ml in the presence of KLB (200 ng/ml). The images were chosen based on three independent experiments (n = 3). Orange arrows indicated individual mitochondrion. **b** Quantification of mitochondrial number (per cell) in chondrocytes induced by FGF19 at 200 ng/ml in the presence of KLB (200 ng/ml). Quantitative analyses of the mitochondrial number were based on nine cells (per group) from three independent experiments (n = 3). **c** Representative immunofluorescent staining showing the number changes of mitochondria in living chondrocytes induced by FGF19 (200 ng/ml) in the presence of KLB (200 ng/ml) for 72 h. The images were chosen based on three independent experiments (n = 3). Red, individual mitochondrion; Green, F-actin; Blue, nucleus. **d** Linear quantification of fluorescence intensity of mitochondrion number in chondrocytes induced by FGF19 at 200 ng/ml in the presence of KLB (200 ng/ml) by Image Pro Plus 6.0. **e** ATP assay showing the increase of intracellular ATP products in chondrocytes induced by FGF19 at 200 ng/ml in the presence of KLB (200 ng/ml). The results were based on three independent experiments (n = 3). **f** Representative western blotting showing the expression change of CS in chondrocytes induced by FGF19 at 200 ng/ml in the presence of KLB (200 ng/ml). The images were chosen based on three independent experiments (n = 3). **g** Quantification of CS by western blotting in (**f**). The data in **b** are shown as box (from 25, 50 to 75%) and whisker (minimum to maximum values) plots. The significant difference analysis in **b**, **e** and **g** was based on Student T-test

To explore the influence of FGF19 on the biological behaviors of mitochondria, we first used TEM to observe the morphological change of mitochondria in chondrocytes induced by FGF19 with the help of β Klotho (KLB), a vital accessory transmembrane glycoprotein for assisting the binding of FGF19 to its receptor [20]. We found that FGF19 at 200 ng/ml could significantly increase the mitochondrial biogenesis as indicated in Fig. 1a. Quantification confirmed that the number of mitochondria in chondrocytes in the FGF19 + KLB group was significantly enhanced relative to that of the single FGF19 group or the KLB control group (Fig. 1b). To further confirm the number change of mitochondria in living chondrocytes, we then used mitochondria staining kit for living cells (Cell Navigator™ Mitochondrion Staining) and performed immunofluorescence. The results revealed that the mitochondria number was significantly enhanced in FGF19-treated living chondrocytes in the presence of KLB (Fig. 1c). Linear quantification of fluorescence intensity (red) also showed that the number of mitochondria was increased and the distribution of mitochondria was broader in the cytoplasm region of living chondrocytes by FGF19 (Fig. 1d). The increase of mitochondrial biogenesis is usually accompanied with the generation of ATP products [30]. Thus, intracellular ATP products were tested by enhanced ATP assay kit in chondrocytes induced by FGF19 at 200 ng/ml in the presence of KLB (200 ng/ml) at 48 h. Results confirmed that the intracellular ATP products in chondrocytes were considerably increased by FGF19 (Fig. 1e). By using western blotting, we detected the expression of citrate synthase (CS), one of the key enzymes of aerobic respiration in mitochondria, was up-regulated in chondrocytes induced by FGF19 (200 ng/ml) in the presence of KLB (200 ng/ml) (Fig. 1f, g). Taking together, these results indicated that FGF19 could increase the mitochondrial biogenesis and thus promote energy generation.

### FGF19-induced mitochondrial biogenesis accompanies with a fusion of mitochondria

**Fig. 2:**
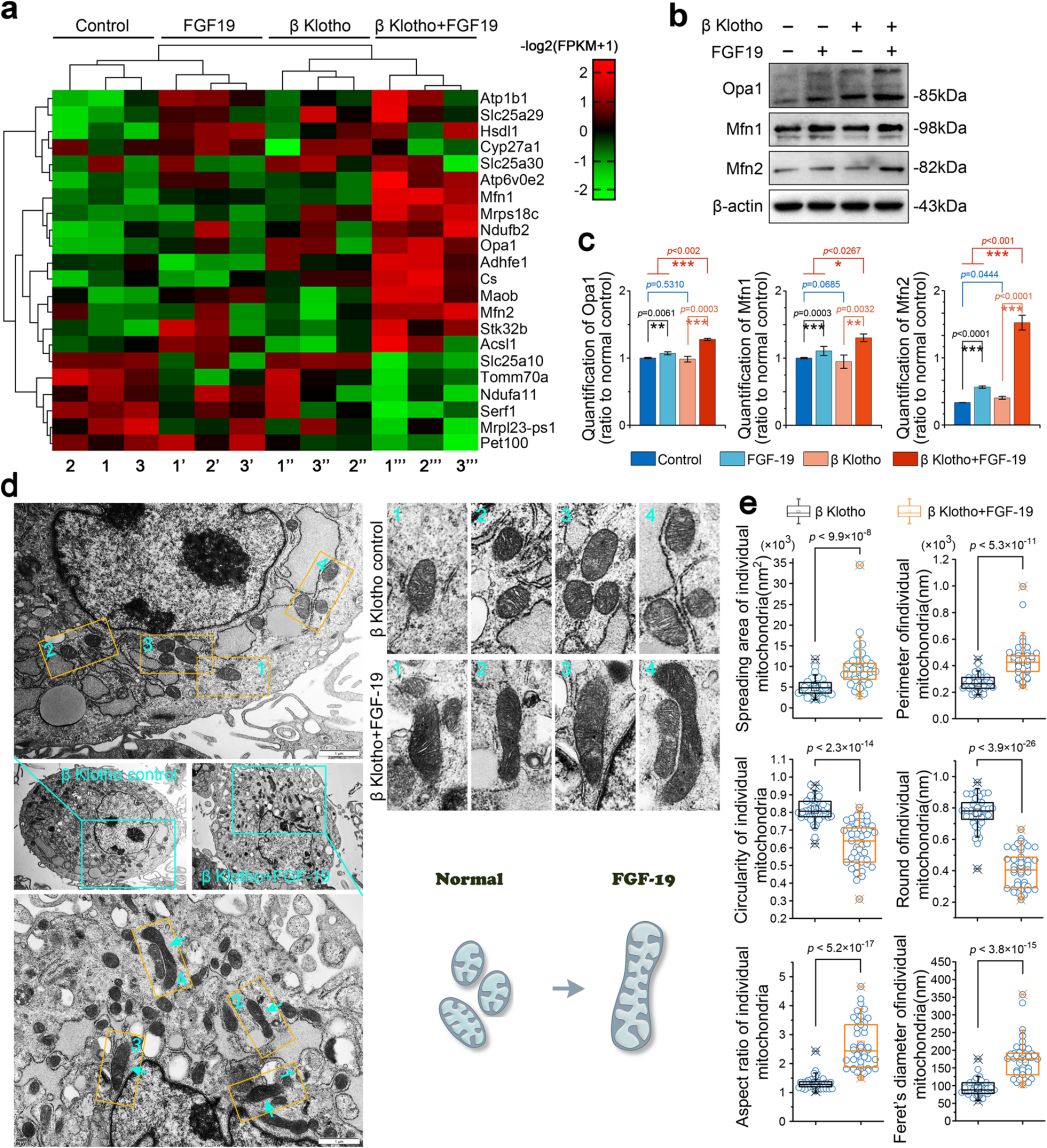
FGF19 promotes the elongation of mitochondrial morphology by up-regulating the expression of mitochondrial fusion proteins. **a** RNA sequencing showing the change of mitochondrial metabolism-related genes in chondrocytes induced by FGF19 at 200 ng/ml in the presence of KLB (200 ng/ml). Three pairs of samples were obtained from three independent cell isolates (n = 3), namely, samples 1, 1′, 1′′and 1′′′, samples 2, 2′, 2′′and 2′′′, and samples 3, 3′, 3′′and 3′′′. The data were present as log2(FPKM + 1). FPKM, Fragments per kilobase of exon model per million mapped fragments. **b** Representative western blotting showing the expression changes of Opa1, Mfn1 and Mfn2 in chondrocytes induced by FGF19 at 200 ng/ml in the presence of KLB (200 ng/ml). The images were chosen based on three independent experiments (n = 3). **c** Quantification of Opa1, Mfn1 and Mfn2 by western blotting in **b** was performed to confirm these protein changes (n = 3). **d** Representative TEM images showing the changes of mitochondrial network’s morphology in chondrocytes induced by FGF19 at 200 ng/ml in the presence of KLB (200 ng/ml). The images were chosen based on three independent experiments (n = 3). Cyan arrows indicated the elongation of mitochondrial morphology. Schematic diagram illustrated that elongation was correlated with mitochondrial fusion. **e** Measurements of mitochondrial network’s morphology in **d** by Image J. Quantitative analyses of mitochondrial network’s morphology were based on three independent experiments (n = 3). The data in **e** were shown as box (from 25, 50 to 75%) and whisker (minimum to maximum values) plots. The significant difference analysis in **c** and **e** was based on Student T-test

We performed RNA sequencing to precisely explore the associated gene changes in mitochondrial metabolism of chondrocytes induced by FGF19 at 200 ng/ml in the presence of KLB (200 ng/ml). Genes shown in red are upregulated and genes in green are downregulated in the pheatmap (Fig. 2a and gene information in Additional file 1: Table S1). We analyzed the expression of all changed genes and screened 22 changed mitochondrion-related genes in chondrocytes induced by FGF19 in the presence of KLB. Among them, mitochondrial fusion genes, Mfn1, Mfn2 and Opa1, were substantially upregulated, which indicates that FGF19 enhances the expression of mitochondrial fusion genes in chondrocytes. By using western blotting, we then confirmed the protein changes of Mfn1, Mfn2 and Opa1 in chondrocytes induced by FGF19 at 200 ng/ml in the presence of KLB (200 ng/ml) at 72 h. As shown in Fig. 2b, FGF19 significantly upregulated the expression of Mfn1, Mfn2 and Opa1 in chondrocytes. Quantitative analysis confirmed the significant increase in mitochondrial-fusion proteins in chondrocytes induced by FGF19 (Fig. 2c). Since mitochondrial fission–fusion is a dynamic process [4], we also detected the protein expression of mitochondrial fission-related proteins, Drp1 and Fis1, by western blotting in chondrocytes induced by FGF19 at 200 ng/ml in the presence of KLB (200 ng/ml) (Additional file 1: Figure S1). Results showed that FGF19 did not significantly change the expressions of Drp1 and Fis1 in chondrocytes induced by FGF19 at 200 ng/ml in the presence of β-Klotho (200 ng/ml). We next used TEM to explore the fission–fusion change of mitochondrial morphology in chondrocytes induced by FGF19 at 200 ng/ml in the presence of KLB (200 ng/ml). The results showed that FGF19 could elongate the individual mitochondrial morphology in chondrocytes (Fig. 2d), especially, the boxed images in yellow showing the elongation of mitochondrial morphology in chondrocytes induced by FGF19. The schematic diagram showed the morphological changes of individual mitochondria transferred from regularly shaped and circular to irregular and elongated. Further, we analyzed the mitochondrial morphology with Image J (Fig. 2e). Quantitative results confirmed a significant increase in spreading area (in nm^2^), perimeter in 2D (in nm), aspect ratio (major to minor axis) and Feret’s diameter (longest distance in one single mitochondrion) of individual mitochondria and a significant decrease of circularity (rated by 4π × area/perimeter^2^) and roundness (rated by 4 × area/π × major axis^2^) of individual mitochondria in chondrocytes induced by FGF19. Together, FGF19 could also mediate fission–fusion process of mitochondria by characterizing the enhancement of fusion proteins and elongation of mitochondrial morphology.

### FGF19 enhances the mitochondrial biogenesis and fusion via up-regulation of AMPKα signaling.

**Fig. 3:**
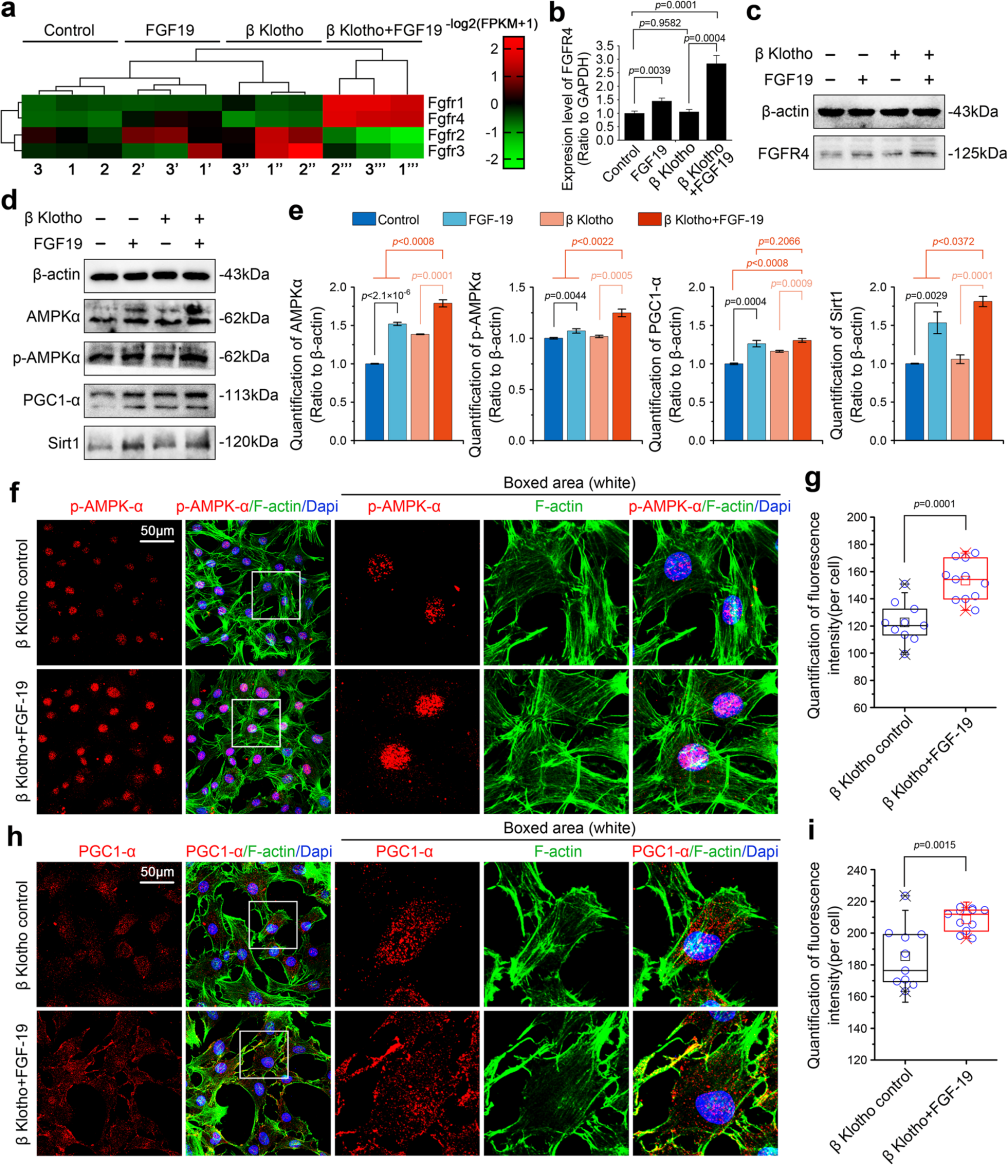
FGF19 increases the mitochondrial biogenesis by up-regulating the expression of AMPKα signalling related proteins in chondrocytes. **a** RNA sequencing showing the change of FGFRs genes in chondrocytes induced by FGF19 at 200 ng/ml in the presence of KLB (200 ng/ml). Three pairs of samples were obtained from three independent cell isolates (n = 3), namely, samples 1, 1′, 1′′and 1′′′, samples 2, 2′, 2′′and 2′′′, and samples 3, 3′, 3′′and 3′′′. The data were present as log2(FPKM + 1). **b** q-PCR showing the gene changes of FGFR4 in chondrocytes induced by FGF19 at 200 ng/ml in the presence of KLB (200 ng/ml). The results were based on three independent experiments (n = 3). **c** Representative western blotting showing the expression change of FGFR4 in chondrocytes induced by FGF19 at 200 ng/ml in the presence of KLB (200 ng/ml). The images were chosen based on three independent experiments (n = 3). **d** Representative western blotting showing the expression changes of AMPKα, p-AMPKα, PGC-1α and SIRT1 in chondrocytes induced by FGF19 at 200 ng/ml in the presence of KLB (200 ng/ml). The images were chosen based on three independent experiments (n = 3). **e** Quantifications of AMPKα, p-AMPKα, PGC-1α and SIRT1 by western blotting in (**d**). f Representative immunofluorescent staining showing the change in the distribution of p-AMPKα in chondrocytes induced by FGF19 (200 ng/ml) in the presence of KLB (200 ng/ml) for 72 h. The images were chosen based on three independent experiments (n = 3). Red, p-AMPKα; Green, F-actin; Blue, nucleus. **g** Quantification of fluorescence intensity of p-AMPKα in chondrocytes induced by FGF19 at 200 ng/ml in the presence of KLB (200 ng/ml). The data were based on at least eight cells from three independent experiments. **h** Representative immunofluorescent staining showing the change in the distribution of PGC-1α in chondrocytes induced by FGF19 (200 ng/ml) in the presence of KLB (200 ng/ml) for 72 h. The images were chosen based on three independent experiments (n = 3). Red, PGC-1α; Green, F-actin; Blue, nucleus. **i** Quantification of fluorescence intensity of PGC-1α in chondrocytes induced by FGF19 at 200 ng/ml in the presence of KLB (200 ng/ml). The data were based on at least eight cells from three independent experiments. The data in **g** and **i** were shown as box (from 25, 50 to 75%) and whisker (minimum to maximum values) plots. The significant difference analysis in **b**, **e**, **g** and **i** was based on Student T-test

It is widely recognized that FGF19 can bind to FGFR1, 2, 3 and 4 receptors but has a high affinity for FGFR4 with the help of KLB [20]. In order to explore the gene expressions of FGFRs in chondrocytes induced by FGF19 at 200 ng/ml in the presence of KLB (200 ng/ml), we analyzed RNA sequencing and the results were shown in the form of pheatmap (Fig. 3a and gene information in Additional file 1: Table S2). The gene expression of FGFR1 and FGFR4 were significantly increased by FGF19 in the presence of KLB, and moreover, the expression of FGFR4 was much higher than that of FGFR1 in the chondrocytes. Then, we performed qPCR and western blotting to affirm the change of FGFR4 expression in chondrocytes induced by FGF19 at 200 ng/ml in the presence of KLB (200 ng/ml). The results in Fig. 3b showed that FGF19 could significantly increase the gene expression of FGFR4 in chondrocytes by qPCR and the up-regulation of FGFR4 gene in the FGF19 + KLB group was remarkably enhanced relative to that without the help of KLB (the single FGF19 group). The protein expression of FGFR4 was also increased in chondrocytes induced by FGF19 (Fig. 3c). With the help of KLB, FGFR4 in the FGF19 + KLB group showed a higher expression than that in the single FGF19 group.

AMPKα signalling directly regulates the biogenesis of mitochondria through the AMPKα-PGC-1α-SIRT1 axis, a putative mitochondrial biogenesis relevant signalling [30]. From western blotting, we found that the expression of AMPKα, p-AMPKα, PGC-1α and SIRT1 was up-regulated in chondrocytes by FGF19 (Fig. 3d). Quantitative analysis of these proteins further confirmed the increase in AMPKα-PGC-1α-SIRT1 signalling in chondrocytes induced by FGF19 in the presence of KLB (Fig. 3e). As the phosphorylation of AMPKα and activation of PGC-1α play a vital role in mitochondrial biogenesis. We further performed immunofluorescent staining to explore the expression and distribution of p-AMPKα and PGC-1α in chondrocytes induced by FGF19 (Fig. 3f–i). The results showed that FGF19 could increase the expression of p-AMPKα and PGC-1α. The expression of p-AMPKα was notably accumulated in the nuclear region (Fig. 3f) while the expression of PGC-1α was increased in whole cytoplasm of chondrocytes (Fig. 3h). Quantification of fluorescent intensity (per cell) confirmed the increase of p-AMPKα and PGC-1α in chondrocytes induced by FGF19 in the presence of KLB (Fig. 3g, i). Taking together, these results indicated that FGF19 could enhance the biogenesis and fusion by up-regulation of AMPKα signalling.

### FGF19 enhances mitochondrial biogenesis and fusion through p38/MAPK pathway

**Fig. 4:**
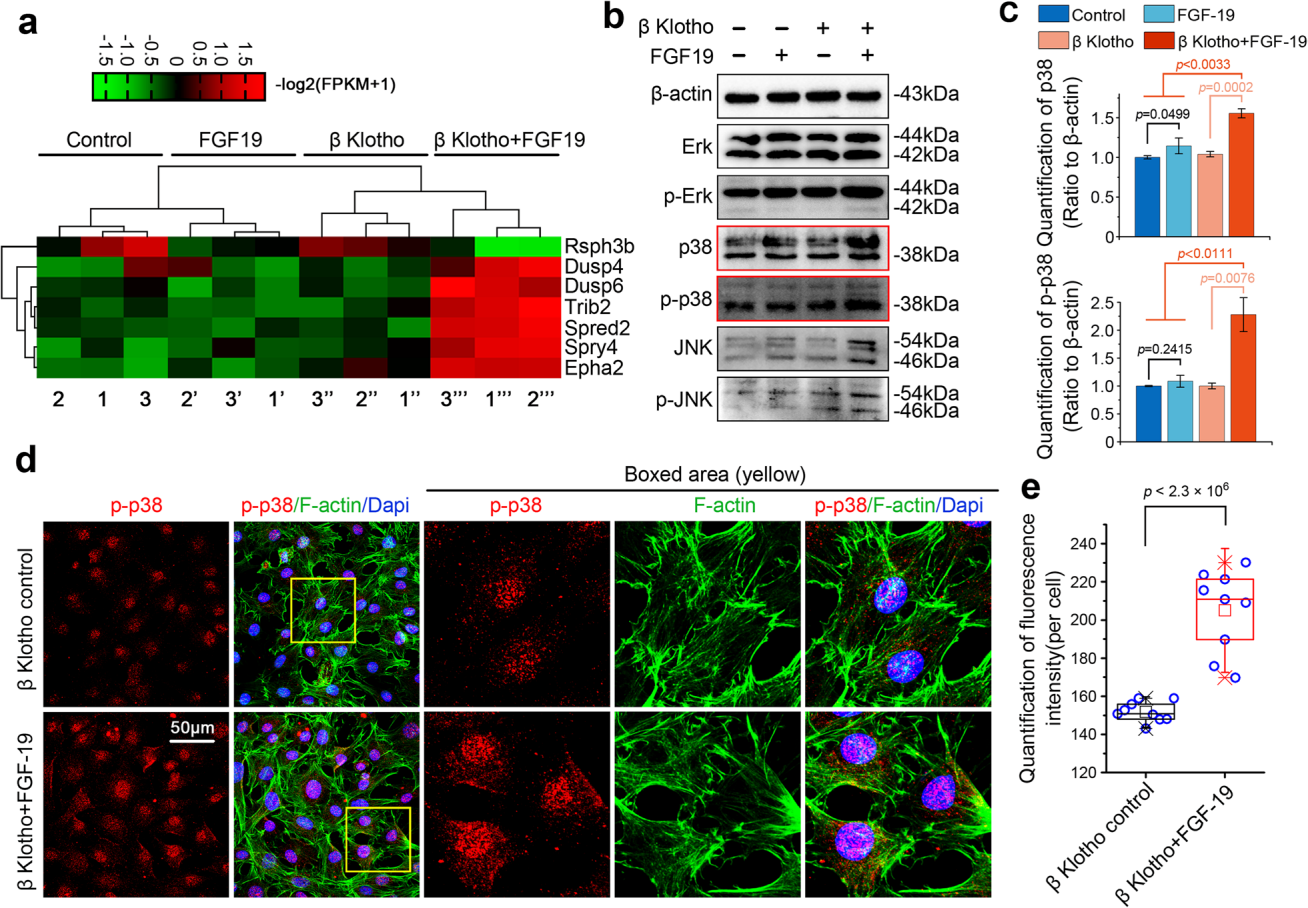
FGF19 activates p38/MAPK signalling in chondrocytes. **a** RNA sequencing showing the changes in the expression of MAPK-related mediators in chondrocytes induced by FGF19 at 200 ng/ml in the presence of KLB (200 ng/ml). Three pairs of samples were obtained from three independent cell isolates (n = 3), namely, samples 1, 1′, 1′′and 1′′′, samples 2, 2′, 2′′and 2′′′, and samples 3, 3′, 3′′and 3′′′. The data were present as log2(FPKM + 1). **b** Representative western blotting showing the expression change of ERK, p-ERK, p38, p-p38, JNK and p-JNK in chondrocytes induced by FGF19 at 200 ng/ml in the presence of KLB (200 ng/ml). The images were chosen based on three independent experiments (n = 3). **c** Quantification of p38 and p-p38 by western blotting in (**b**). **d** Representative immunofluorescent staining showing the change in the expression and distribution of p-p38 in chondrocytes induced by FGF19 (200 ng/ml) in the presence of KLB (200 ng/ml) for 72 h. The images were chosen based on three independent experiments (n = 3). Red, p-p38; Green, F-actin; Blue, nucleus. **e** Quantification of fluorescence intensity of p-p38 in chondrocytes induced by FGF19 at 200 ng/ml in the presence of KLB (200 ng/ml). The data were based on nine cells from three independent experiments. The data in **e** were shown as box (from 25, 50 to 75%) and whisker (minimum to maximum values) plots. The significant difference analysis in **c** and **e** was based on Student T-test

To determine the key cytoplasmic pathways related to FGF19-mediated mitochondrial biogenesis and fusion, we analyzed the RNA sequencing data and screened out all changed kinases involving classical pathways. These kinases were clustered by pheatmap (Fig. 4a and gene information in Additional file 1: Table S3). It showed that most of the kinases were related to MAPK signaling. In particular, MAP kinases such as Dusp4 and Dusp2 were shown to be significantly enhanced in chondrocytes. We then performed western blotting to confirm the changes of ERK/p-ERK, p38/p-p38 and JNK/p-JNK in chondrocytes induced by FGF19 (Fig. 4b). Among them, we found that the enhancement of total p38 and p-p38 were higher than the other two. Quantitative analysis confirmed a significant increase in total p38 and p-p38 but the increase of ERK/p-ERK and JNK/p-JNK was not as obvious as p38/p-p38 in chondrocytes induced by FGF19 in the presence of KLB (Fig. 4c and Additional file 1: S2). We further used immunofluorescent staining to explore the expression and distribution of p-p38 in chondrocytes induced by FGF19 in the presence of KLB (Fig. 4d, e). From the CLSM images, we found that FGF19 could increase the expression of p-p38 in the cytoplasm of chondrocytes, especially in the nuclear region (Fig. 4d). Quantification of total fluorescent intensity confirmed the increased expression of p-p38 in chondrocytes induced by FGF19 in the presence of KLB (Fig. 4e).

### Inhibition of p38/MAPK attenuates AMPKα signalling and impairs the biogenesis and fusion of mitochondria induced by FGF19

**Fig. 5:**
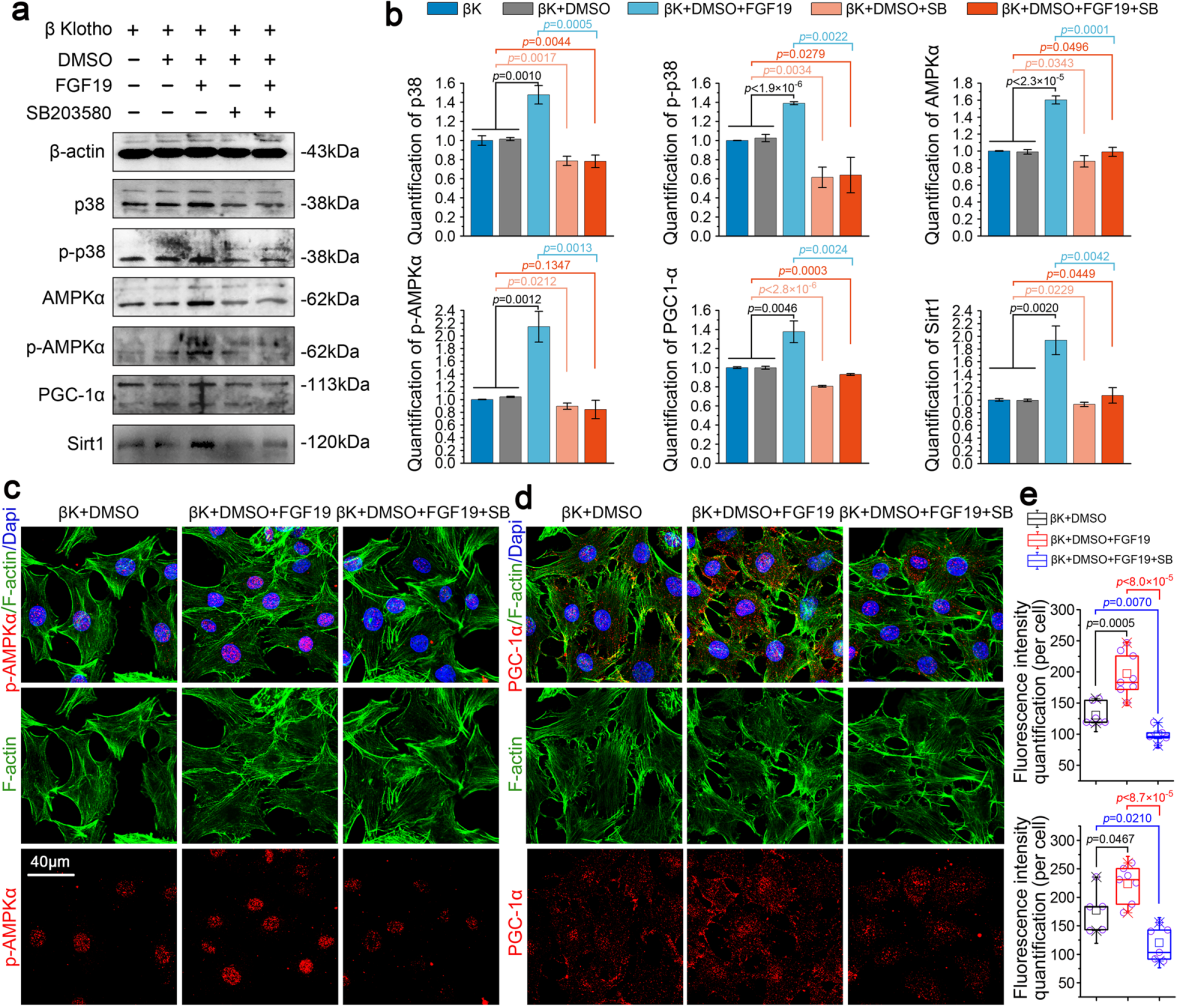
Inhibition of p38 attenuated FGF19-enhanced AMPKα activity. **a** Representative western blotting showing the expression change of p38, p-p38, AMPKα, p-AMPKα, PGC-1α and SIRT1 in chondrocytes induced by SB203580 (10 µM) in the presence of FGF19 (200 ng/ml) and KLB (200 ng/ml). The images were chosen based on three independent experiments (n = 3). **b** Quantification of p38, p-p38, AMPKα, p-AMPKα, PGC-1α and SIRT1 by western blotting in (**a**). **c** Representative immunofluorescent staining showing the change in the distribution of p-AMPKα in chondrocytes induced by SB203580 (10 µM) in the presence of FGF19 (200 ng/ml) and KLB (200 ng/ml). The images were chosen based on three independent experiments (n = 3). Red, p-AMPKα; Green, F-actin; Blue, nucleus. **d** Representative immunofluorescent staining showing the change in the expression and distribution of PGC-1α in chondrocytes induced by SB203580 (10 µM) in the presence of FGF19 (200 ng/ml) and KLB (200 ng/ml). The images were chosen based on three independent experiments (n = 3). Red, PGC-1α; Green, F-actin; Blue, nucleus. **e** Quantification of fluorescence intensity of p-AMPKα and PGC-1α in chondrocytes induced by SB203580 (10 µM) in the presence of FGF19 (200 ng/ml) and KLB (200 ng/ml). The data in **e** were shown as box (from 25, 50 to 75%) and whisker (minimum to maximum values) plots. The significant difference analysis in **b** and **e** was based on Student T-test

To further determine the importance of p38/MAPK in regulating the expression of AMPKα signalling, SB203580, a specific inhibitor of p38/p-p38 signalling, was utilized [31]. We detected the expressions of p38/p-p38, AMPKα/p-AMPKα, PGC-1α and Sirt1 in chondrocytes induced by FGF19 (200 ng/ml) in the presence of KLB (200 ng/ml) after pretreatment of SB203580 (10 µM) for 2 h (Fig. 5a). From western blotting, we revealed that SB203580 could effectively impair the up-regulation of p38/p-p38 and also attenuated the mitochondrial biogenesis proteins including AMPKα/p-AMPKα, PGC-1α and Sirt1. Quantitative analysis further confirmed a significant decrease in the expression of p38 pathway and AMPKα signalling in chondrocytes induced by FGF19 (200 ng/ml) in the presence of KLB (200 ng/ml) after pretreatment of SB203580 (10 µM) (Fig. 5b). We then used immunofluorescent staining to show the expression and distribution of p-AMPKα and PGC-1α (Fig. 5c–e). The results showed that the expressions of p-AMPKα (Fig. 5c) and PGC-1α (Fig. 5d) were largely reduced in chondrocytes induced by FGF19 (200 ng/ml) in the presence of KLB (200 ng/ml) after pretreatment of SB203580 (10 µM). Fluorescence quantification further confirmed the changes of p-AMPKα and PGC-1α (Fig. 5e).

**Fig. 6:**
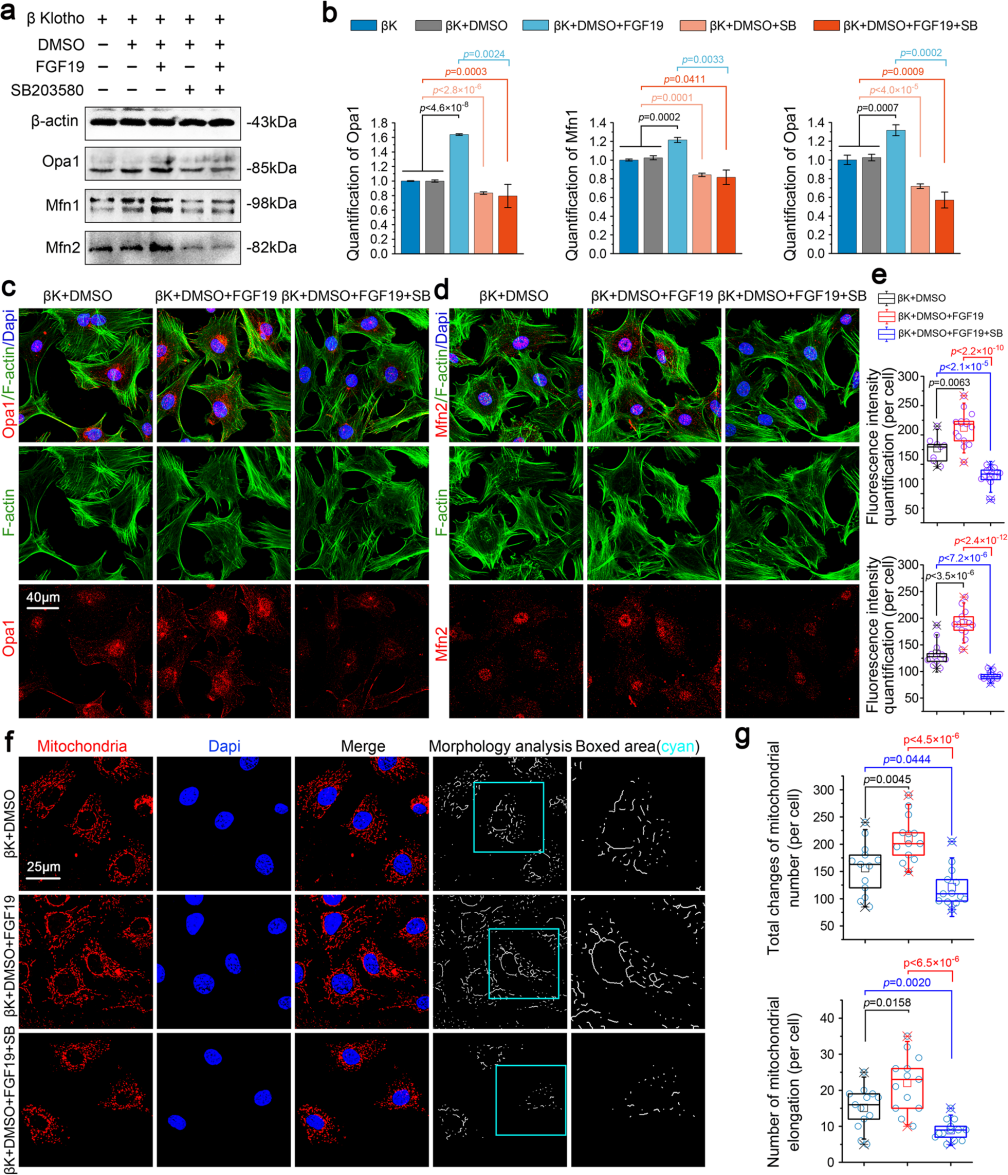
Inhibition of p38 decreases the expressions of mitochondrial fusion proteins induced by FGF19 in chondrocytes. **a** Representative western blotting showing the expression change of Opa1, Mfn1 and Mfn2 in chondrocytes induced by SB203580 (10 µM) in the presence of FGF19 (200 ng/ml) and KLB (200 ng/ml). The images were chosen based on three independent experiments (n = 3). **b** Quantification of Opa1, Mfn1 and Mfn2 by western blotting in (**a**). **c** Representative immunofluorescent staining showing the change in the expression and distribution of Opa1 in chondrocytes induced by SB203580 (10 µM) in the presence of FGF19 (200 ng/ml) and KLB (200 ng/ml). The images were chosen based on three independent experiments (n = 3). Red, Opa1; Green, F-actin; Blue, nucleus. **d** Representative immunofluorescent staining showing the change in the distribution of Mfn2 in chondrocytes induced by SB203580 (10 µM) in the presence of FGF19 (200 ng/ml) and KLB (200 ng/ml). The images were chosen based on three independent experiments (n = 3). Red, Mfn2; Green, F-actin; Blue, nucleus. **e** Quantification of fluorescence intensity of Opa1 and Mfn2 in chondrocytes induced by SB203580 (10 µM) in the presence of FGF19 (200 ng/ml) and KLB (200 ng/ml). The data were based on at least eight cells from three independent experiments. **f** Representative immunofluorescent staining showing the changes of morphology mitochondrial network in living chondrocytes induced by SB203580 (10 µM) in the presence of FGF19 (200 ng/ml) and KLB (200 ng/ml) for 72 h. Image J shows the change of mitochondrial network morphology analysis in cyan boxes. The images were chosen based on three independent experiments (n = 3). Red, mitochondrial network; Blue, nucleus. **g** Quantification of mitochondrial number (per cell) and mitochondrial elongated number (per cell) in chondrocytes induced by SB203580 (10 µM) in the presence of FGF19 (200 ng/ml) by Image J. Quantitative analyses were based on three independent experiments (n = 3). The data in **e** and **g** were shown as box (from 25, 50 to 75%) and whisker (minimum to maximum values) plots. The significant difference analysis in **b**, **e** and **g** were based on Student T-test

Next, we explored the role of p38/MAPK in regulating biogenesis and fusion of mitochondria. We detected the protein mediators in the fission–fusion process (Additional file 1: Figure S3 and 6a). The results showed that inhibition of p38 did not significantly change the expression of FGF19-induced mitochondrial fission proteins, i.e., Drp1 and Fis1 (Additional file 1: Figure S3), but indeed decreased the expression of FGF19-induced mitochondrial fusion proteins, i.e., Opa1, Mfn1 and Mfn2 (Fig. 6a, b). Further, we performed immunofluorescence and found the impairment of Opa1 (Fig. 6c) and Mfn2 (Fig. 6d) in chondrocytes induced by FGF19 (200 ng/ml) in the presence of KLB (200 ng/ml) after pretreatment of SB203580 (10 µM). Fluorescence quantification further confirmed the changes of Opa1 and Mfn2 (Fig. 6e).

Finally, to confirm the role of p38/MAPK in controlling mitochondrial network morphology, we applied mitochondrial living cell staining (Fig. 6f, g). From CLSM, we observed that SB203580 could significantly decrease the FGF19-enhanced mitochondrial number, and moreover, it could sharply reduce the mitochondrial network morphology formed by FGF19 (cyan boxes). Quantitative analysis confirmed that about a 50% decrease in the total change of mitochondrial number (per cell) and a 60% decrease in the number of mitochondrial elongation (per cell) in chondrocytes induced by SB203580 (Fig. 6g).

Together, the inhibition of p38/MAPK could decrease the expression of AMPKα signalling, and thus impair the biogenesis and fusion of mitochondria by characterizing mitochondrial fusion proteins, and mitochondrial network morphology in living chondrocytes.

## Discussion

It has been well established that cartilage is an avascular, non-lymphatic, and non-innervated tissue composed of ECM and chondrocytes [32]. Chondrocytes, the only mature cell type, in cartilage are surrounded by a relatively low-oxygen extracellular environment. In chondrocytes, glycolysis and OXPHOS are both present, and the ATP produced by OXPHOS makes up about 25% of the total energy in chondrocytes [23]. Nonetheless, reports have confirmed that OXPHOS is a kind of more effective method of ATP synthesis [33]. OXPHOS thus plays a significant role in the energy metabolism of chondrocytes. Four ETC complexes (respiratory chain complexes I–IV) and complex V (ATP synthase) of the respiratory chain, which is located on the IMM, produce ATP during the OXPHOS process [34]. Mitochondrial dysfunction impairs ATP generation and further interferes with the repair process against cartilage degradation [35]. For these reasons, mitochondria are indispensable energy-producing organelles in the OXPHOS of chondrocytes. In this study, we discovered that FGF19 could greatly boost the production of the key enzyme, CS, for aerobic respiration, as well as intracellular ATP products (Fig. 1e–g). Our findings also showed that FGF19 might increase mitochondrial biogenesis by enhancing the number of functional mitochondria in chondrocytes (Fig. 1a–d). These results suggest that FGF19 is involved in mitochondrial biogenesis and fusion and enlarge our understanding of chondrocyte metabolism induced by growth factors.

FGFs family members, in particular the members of the FGF19 subfamily, are a type of cytokines that play an important role in regulating cellular energy homeostasis and mitochondrial function [36]. Previous researches indicated that FGF19 played a pivotal role in glucose metabolism. According to reports, FGF19 not only improves hepatic gluconeogenesis and glucose catabolism by activating the CREB-PGC-1α signalling cascade pathway, but also enhances glycogen synthesis by increasing glycogen synthetase (GS) activity [16]. Martinez et al. also found that FGF19 levels are correlated with the mitochondrial number in white adipose tissue [17]. In addition to FGF19, the FGF19 subfamily also includes FGF21 and FGF23. FGF21 was reported as a biomarker with high sensitivity for predicting mitochondrial disease in muscle [37]. Moreover, deletion of the fission-related protein DRP1 from the mice liver disrupted mitochondrial fission, which would further promote the expression of FGF21 [38]. Furthermore, it was discovered that FGF21 activates the AMPK-SIRT1-PGC-1α pathway to regulate mitochondrial fission–fusion, increase mitochondrial biogenesis, and promote mitochondrial function [39]. Another FGF19 subfamily member, FGF23, could enhance mitochondrial function by upregulating CS activity [40]. FGF23 treatment increased peroxisome proliferator-activated receptor δ (PPAR-δ) mRNA levels and improved mitochondrial function. Other FGF subfamily members may also affect mitochondrial function. For instance, it has been suggested that FGF13 may improve mitochondrial function in primary cortical neurons [41]. Interestingly, we also found that FGF19 could significantly change the mitochondrion-related gene expression in chondrocytes (Fig. 2a). The regulation of chondrocyte mitochondria by FGF19 extends our understanding of FGF19. Since FGF21 and FGF23 are both FGF19 subfamily members, we are interested in whether the same changes in mitochondria will occur by the induction of FGF19. The mitochondrial metabolism induced by FGF19 may also be similar in other subfamily members. More detailed studies are needed to confirm this assumption.

The mitochondrial network keeps the proper balance between fission and fusion, which helps to maintain dynamic homeostasis of mitochondrial biogenesis [42]. In addition to controlling mitochondrial biogenesis, fission and fusion proteins may also control mitochondrial bioenergetics. Traditionally, mitofusins drive the fusion of outer mitochondrial membrane and regulate the shape of mitochondrial cristae structure [43]. However, it is reported that the master regulator effect of PGC-1 on mitochondrial biogenesis may require or may be mediated by Mfn2. Mfn2 overexpression activated mitochondrial metabolism by increasing the expression of several subunits of OXPHOS complexes in muscle cells and the connection between Mfn2 and mitochondrial metabolism has been also demonstrated using loss-of-function studies [44]. The function of Mfn2 in mitochondrial energy metabolism was also demonstrated in Mfn2 knockdown mouse embryonic fibroblasts that Mfn2 affects mitochondrial energy metabolism by inhibiting the expression of complexes I, II, III and V and reducing mitochondrial membrane potential [45]. The deletion of Mfn2 causes a deficiency in coenzyme Q that leads to electron transport chain (ETC) dysfunction and a decrease in ATP production. Opa1 resides and works in the IMM after the Mfn1/2 proteins are anchored in the OMM. In general, the crucial determinants of bioenergetic efficiency depend on the cristae structure on the IMM. The change in the morphology of mitochondria is inevitably related to IMM remodeling. OPA1 inactivation significantly alters the mitochondrial morphology, resulting in scattered mitochondrial fragments and disordered mitochondrial cristae [46]. On the other hand, OPA1 overexpression can favor the assembly and stability of respiratory chain supercomplexes (RCS) by changing cristae shape [47]. The change between these mitochondrial fission–fusion proteins and mitochondrial biogenesis in chondrocytes was the main focus of the current work. And we found that mitochondrial fusion-related proteins Mfn1, Mfn2 and Opa1 were significantly enhanced by FGF19 (Fig. 2a–c), which was accompanied with mitochondrial biogenesis (Fig. 1). Moreover, elongated mitochondria were reported to have more cristae structure and higher ATP synthase activity [1]. Additionally, mitochondrial fusion could lead to the elongation of the mitochondrial network under physiological conditions [48]. Thus, mitochondria with an elongated morphology are regarded to be more bioenergetically efficient. As the study has demonstrated, FGF19 stimulated the fusional changes of mitochondria in chondrocytes (Fig. 2d, e). We discovered for the first time that FGF19 could upregulate mitochondrial biogenesis and mitochondrial fusion process by regulating fusion-related proteins in chondrocytes and thus promote the elongation of mitochondria in chondrocytes.

**Fig. 7:**
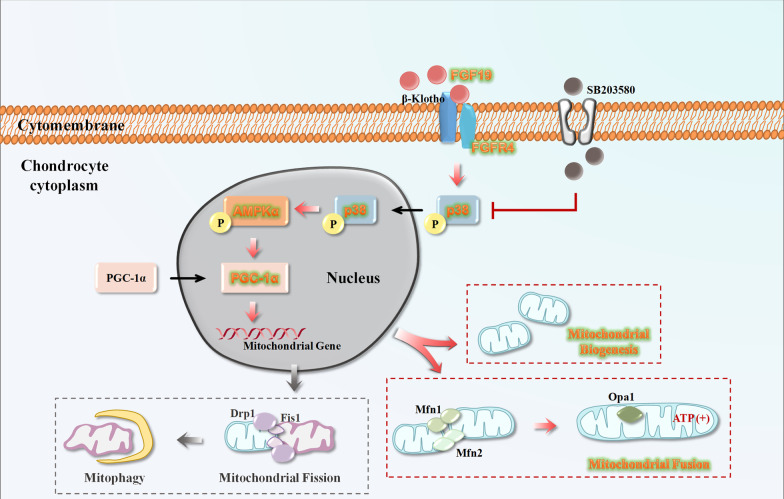
The schematic diagram showing how FGF19 mediates mitochondrial fusion in chondrocytes. In the present study, the result indicates that FGF19 binds to FGFR4, activates the p38/MAPK signaling and AMPKα signaling, and resultantly induces the mitochondrial biogenesis and fusion in chondrocytes

Mitochondria provide energy and are involved in several metabolic activities through various signalling pathways. It is well-known that the AMPK pathway is associated with mitochondria. AMPK could sense the changes in the energy status of cells and adapt mitochondrial function by regulating its biogenesis, MQC and dynamics [30]. Conversely, a deficiency of mitochondrial biogenesis could decrease the phosphorylation of AMPK. In the current study, we provided solid evidence to prove that FGF19 stimulation could enhance mitochondrial biogenesis and fusion via up-regulating AMPKα signalling (Fig. 3d–i). The level of mitochondrial biogenesis may be related to the level of AMPK phosphorylation. As reported that the decline of p-AMPK further leads to the depression of NAD^+^ -dependent deacetylase SIRT-1 and the mitochondrial biogenesis master regulator PGC-1α. The activation of PGC-1α not only leads to its translocation from the cytoplasm to the nucleus, but also upregulates the transcription of genes that are important for mitochondrial OXPHOS [49]. As we found in this study, SIRT-1 and PGC1α expression was also upregulated in chondrocytes by FGF19 (Fig. 3f–i). These results confirm that FGF19 enhances mitochondrial biogenesis and fusion by upregulating AMPKα signalling. It is reported that FGF19 related downstream signalling pathway mainly includes the MAPK, the phosphatidylinositol 3-kinase- (PI3K-) AKT, the phospholipase C (PLC) γ-protein kinase C (PKC), and the signal transducer and activator of transcription (STAT) pathway [50]. Among them, MAPK signalling, as a canonical FGFs family signalling pathway, has been confirmed to be an important downstream pathway in maintaining the homeostasis of cartilage [36]. Studies on the development of craniofacial cartilage in zebrafish have found that estrogen may disrupt the bone-related MAPK signalling pathway by affecting FGF19 [51]. And according to our research, most of the changed kinases involving classical pathways kinases were related to MAPK signalling in chondrocytes induced by FGF19 (Fig. 4a). Hence, MAPK signalling may be a vital pathway in enhancing mitochondrial biogenesis and fusion in chondrocytes with FGF19 treatment. We also discover that FGF19 activated the MAPK subfamilies p-ERK, p-JNK, and p-p38 with p-p38 being the most significant (Fig. 4b, c). Besides, p38 is generally considered an active regulator in chondrogenesis and chondrocyte differentiation [52]. Hence, we also provided evidence to validate mitochondrial biogenesis and fusion process in chondrocytes were mediated by p-38/MAPK signalling pathway. Inhibition of p38/MAPK attenuates AMPKα signaling (Fig. 5) and further impairs the biogenesis and fusion of mitochondria induced by FGF19 (Fig. 6). For these reasons, p-38/MAPK signalling pathway is one of the most important pathways that are activated by FGF19. However, this could not be the only pathway that can modulate mitochondrial fusion by FGF19 since the expression of mitochondrial fusion-related proteins was not completely abrogated by using SB203580. We assume that there may be other signal pathways involved in the regulation of mitochondrial fusion in chondrocytes. It will be interesting to identify which pathways are also involved in the process of mitochondrial fission–fusion by FGF19 in future studies (Fig. 7).

Inflammatory joint diseases, such as osteoarthritis (OA), are characterized by metabolic disorders. In OA, chondrocytes rapidly change their metabolic pathways in the process of OA disease [53]. Therefore, exploring the mechanism of chondrocyte metabolism may provide potential new therapeutic strategies for the treatment of OA and other inflammatory joint diseases. Previous researches have verified that FGFs could cause cell metabolic disorders and work as key participants in morphogenesis, angiogenesis, neoplastic and several diseases [54]. For instance, FGF21 was found to be related to glucose and lipid metabolism [55]. FGF23 was reported to be involved in phosphate and vitamin D metabolism [56]. FGF2 and FGF18 were revealed to participate in cartilage remodeling [57]. And FGF20 was verified to be associated with cartilage pathology [58]. As for FGF19, it was recognized to be an important growth factor in cell metabolism and cartilage development, because it acted as a critical metabolic regulator in bile acid biosynthesis [59], gallbladder filling [60], glucose metabolism [37] and skeletal muscle development [61]. Besides, FGF19 was also reported to play a key role in growth plate development [14] and morphogenesis during craniofacial development [51]. Therefore, exploring the change of FGF19-mediated cellular metabolism in chondrocytes enlarges our understanding of the physiology and pathology of cartilage and chondrocytes.

In summary, we demonstrated that FGF19 promotes the process of mitochondrial fusion and elongates the morphology of mitochondrial network in chondrocytes and revealed the potential mechanism of mitochondrial fusion mediator proteins regulation in chondrocytes. These findings enhance our understanding of the molecular mechanisms of mitochondrial dynamics in chondrocytes and provide a new potential for therapeutic targets for the management of cartilage diseases.

### Supplementary Information

**Additional file 1**. **Figure S1**. FGF19 do not significantly change the expressions of mitochondrial fission-related proteins in chondrocytes. **Figure S2**. Quantitative analysis of western blotting in Fig. 4b indicates that FGF19 induces a higher expression of p-p38 signalling than the other two ones, p-Erk and p-JNK in chondrocytes in the presence of β-Klotho. **Figure S3**. Inhibition of p38 changes the expression of FGF19-induced mitochondrial fission proteins in chondrocytes. **Table S1**. RNA sequencing showing the change of mitochondrial metabolism-related genes in chondrocytes treated with FGF19 at 200 ng/ml in the presence of KLB (200 ng/ml). **Table S2**. RNA sequencing showing the change of FGFRs genes in chondrocytes treated with FGF19 at 200 ng/ml in the presence of KLB (200 ng/ml). **Table S3**. RNA sequencing showing the changes in the expression of MAPK-related mediators in chondrocytes treated with FGF19 at 200 ng/ml in the presence of KLB (200 ng/ml). 

## Data Availability

Any data generated in this study are available from the corresponding author upon request in addition to source data.
