# Correction: FGF19 increases mitochondrial biogenesis and fusion in chondrocytes via the AMPKα-p38/MAPK pathway

**DOI:** 10.1186/s12964-023-01279-x

**Published:** 2023-08-21

**Authors:** Shiyi Kan, Caixia Pi, Li Zhang, Daimo Guo, Zhixing Niu, Yang Liu, Mengmeng Duan, Xiahua Pu, Mingru Bai, Chenchen Zhou, Demao Zhang, Jing Xie

**Affiliations:** 1 Lab of Bone and Joint Disease, State Key Laboratory of Oral Diseases, West China Hospital of Stomatology, Sichuan University, Chengdu, 610064 Sichuan China; 2 National Clinical Research Center for Oral Diseases, West China Hospital of Stomatology, Sichuan University, Chengdu, 610064 China

**Correction: Cell Commun Signal 21, 55 (2023)**

**https://doi.org/10.1186/s12964-023-01069-5**

Following the publication of the original article [1], the authors found an error on SEM images (normal control group) in Fig. 1a. Based on a rigorous attitude, here we have provided the corrected Fig. 1a. This corrected image (normal control) does not affect any conclusion of the article.

**Fig. 1:**
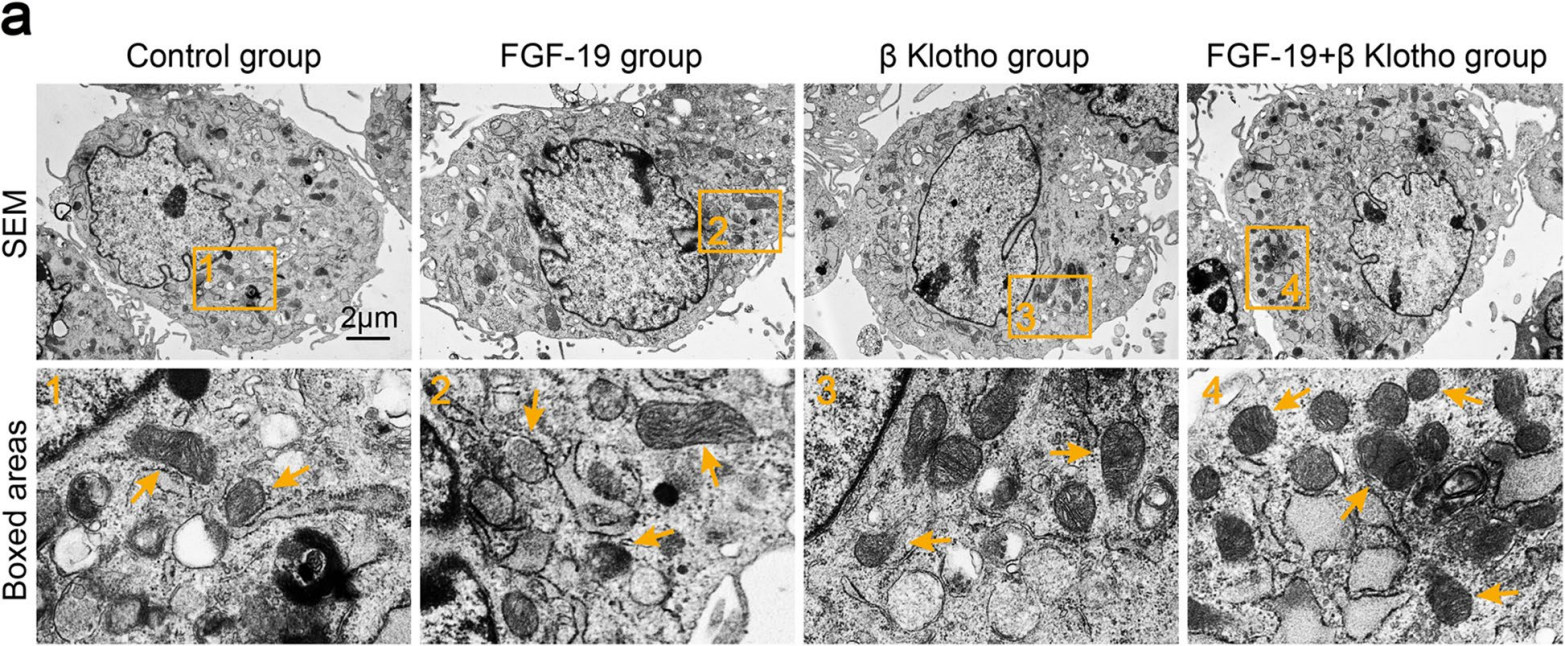
FGF19 induces a transient increase in mitochondrial number and an enhanced generation of ATP products. **a** Representative TEM images showing the changes of mitochondrial number in chondrocytes induced by FGF19 at 200 ng/ml in the presence of KLB (200 ng/ml). The images were chosen based on three independent experiments (*n* = 3). Orange arrows indicated individual mitochondrion

The original article [1] has been corrected. 
