# Supplementary material for: FGF19 increases mitochondrial biogenesis and fusion in chondrocytes via the AMPKα-p38/MAPK pathway
Source: Cell Commun Signal. 2023 Mar 13;21:55. doi: 10.1186/s12964-023-01069-5 (PMC10009974; doi:10.1186/s12964-023-01069-5)
Supplement: Supplementary file 2 — Additional file 1. Figure S1. FGF19 do not significantly change the expressions of mitochondrial fission-related proteins in chondrocytes. Figure S2. Quantitative analysis of western blotting in Fig. 4b indicates that FGF19 induces a higher expression of p-p38 signalling than the other two ones, p-Erk and p-JNK in chondrocytes in the presence of β-Klotho. Figure S3. Inhibition of p38 changes the expression of FGF19-induced mitochondrial fission proteins in chondrocytes. Table S1. RNA sequencing showing the change of mitochondrial metabolism-related genes in chondrocytes treated with FGF19 at 200 ng/ml in the presence of KLB (200 ng/ml). Table S2. RNA sequencing showing the change of FGFRs genes in chondrocytes treated with FGF19 at 200 ng/ml in the presence of KLB (200 ng/ml). Table S3. RNA sequencing showing the changes in the expression of MAPK-related mediators in chondrocytes treated with FGF19 at 200 ng/ml in the presence of KLB (200 ng/ml). [file 12964_2023_1069_MOESM2_ESM.doc]

Supplementary materials for original paper

**FGF19 increases mitochondrial biogenesis and fusion in chondrocytes via the AMPKα-p38/MAPK signalling**

Shiyi Kan, Caixia Pi, Li Zhang, Daimo Guo, Zhixing Niu, Yang Liu, Mengmeng Duan, Xiahua Pu, Mingru Bai, Chenchen Zhou, Demao Zhang, Jing Xie

**1. Supplementary Figures**

**Figure S1.**

**
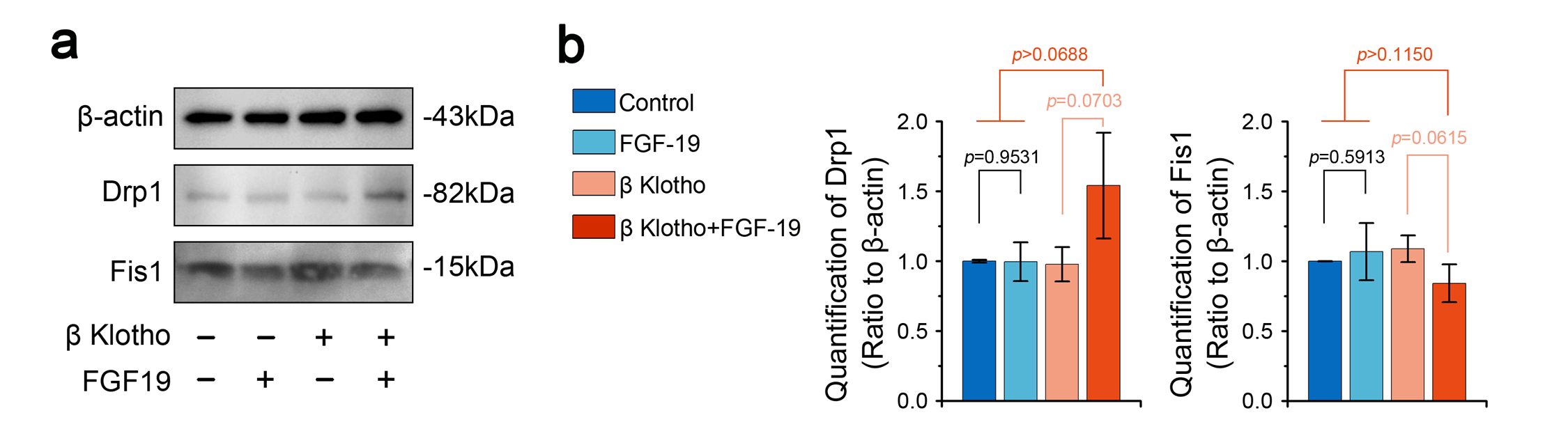
**

**Figure S1. FGF19 do not significantly change the expressions of mitochondrial fission-related proteins in chondrocytes.**

**a.** Representative western blotting showing the expression change of Drp1 and Fis1 in chondrocytes induced by FGF19 at 200 ng/ml in the presence of β-Klotho (200 ng/ml). The images were chosen based on three independent experiments (n = 3).

**b.** Quantification of Drp1 and Fis1 by western blotting in **a**.

The significant differences in **b** were based on Student T-test.

**Figure S2.**

**
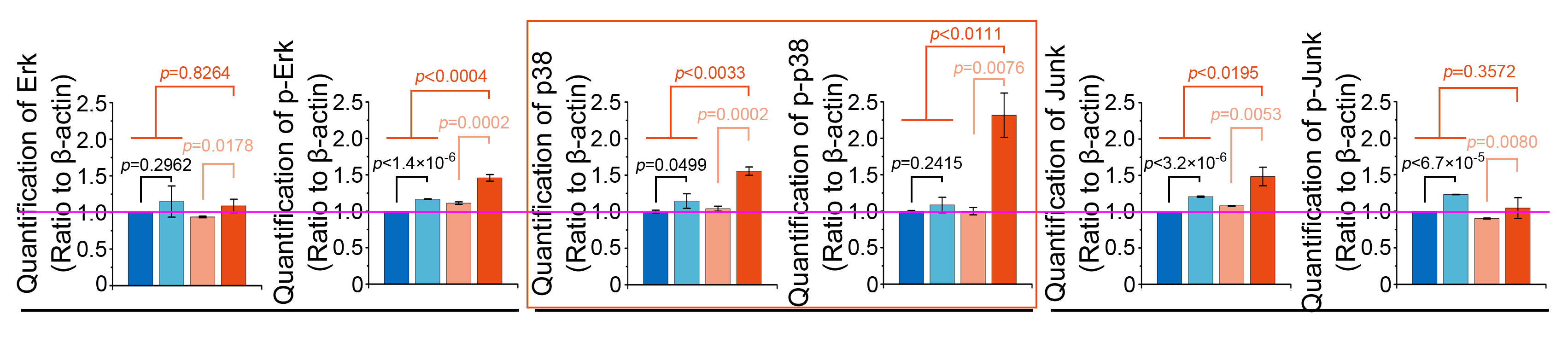
**

**Figure S2.** Quantitative analysis of western blotting in Figure4b indicates that FGF19 induces a higher expression of p-p38 signaling than the other two ones, p-Erk and p-JNK in chondrocytes in the presence of β-Klotho.

**Figure S3.**

**
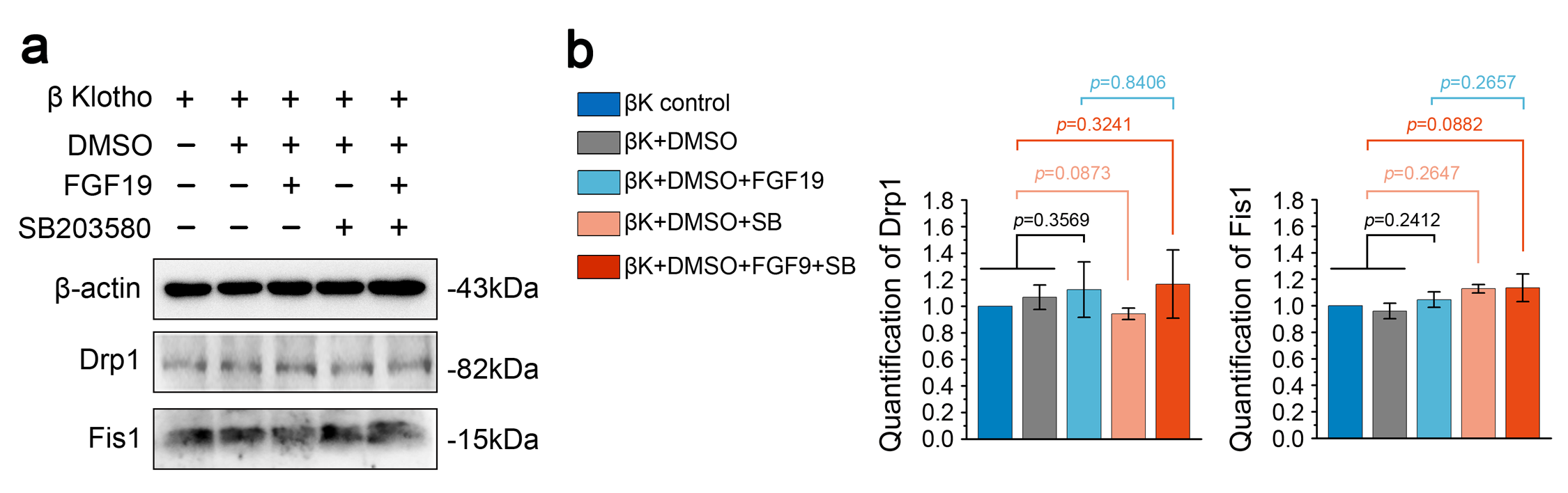
**

**Figure S3. Inhibition of p38 changes the expression of FGF19-induced mitochondrial fission proteins in chondrocytes.**

**a.** Representative western blotting showing the expression change of Drp1 and Fis1 in chondrocytes induced by SB203580 (10 µM) in the presence of FGF19 (200 ng/ml) and β-Klotho (200 ng/ml). The images were chosen based on three independent experiments (n = 3).

**b.** Quantification of Drp1 and Fis1 by western blotting in **a**.

The significant differences in **b** were based on Student T-test.

**2. Supplementary tables**

**Table S1.** RNA sequencing showing the change of mitochondrial metabolism-related genes in chondrocytes induced by FGF19 at 200 ng/ml in the presence of KLB (200 ng/ml). Data were presented as FPKM.

**Table S2.** RNA sequencing showing the change of FGFRs genes in chondrocytes induced by FGF19 at 200 ng/ml in the presence of KLB (200 ng/ml). Data were presented as FPKM.

**Table S3.** RNA sequencing showing the changes in the expression of MAPK-related mediators in chondrocytes induced by FGF19 at 200 ng/ml in the presence of KLB (200 ng/ml). Data were presented as FPKM.
